# Summary of the National Cancer Institute 2023 Virtual Workshop on Medical Image De-identification—Part 1: Report of the MIDI Task Group - Best Practices and Recommendations, Tools for Conventional Approaches to De-identification, International Approaches to De-identification, and Industry Panel on Image De-identification

**DOI:** 10.1007/s10278-024-01182-y

**Published:** 2024-07-12

**Authors:** David Clunie, Fred Prior, Michael Rutherford, Stephen Moore, William Parker, Haridimos Kondylakis, Christian Ludwigs, Juergen Klenk, Bob Lou, Lawrence (Tony) O’Sullivan, Dan Marcus, Jiri Dobes, Abraham Gutman, Keyvan Farahani

**Affiliations:** 1 PixelMed Publishing, Bangor, PA USA; 2 University of Arkansas for Medical Sciences, Little Rock, AR USA; 3 Washington University School of Medicine in St. Louis, St. Louis, MO USA; 4 University of British Columbia, Vancouver, Canada; 5 Institute of Computer Science, Foundation of Research & Technology - Hellas (FORTH), Heraklion, Greece; 6 Aigora GmbH, Munich, Germany; 7 Deloitte Consulting, New York, NY USA; 8 Google, Mountain View, CA USA; 9 IBIS, Princeton, NJ USA; 10 Flywheel, Minneapolis, MN USA; 11 John Snow Labs, Lewes, DE USA; 12 AG Mednet, Boston, MA USA; 13 National Heart, Lung, and Blood Institute, National Institutes of Health, Bethesda, MD USA

**Keywords:** De-identification, Best practices, DICOM, International concerns, Workflow

## Abstract

De-identification of medical images intended for research is a core requirement for data-sharing initiatives, particularly as the demand for data for artificial intelligence (AI) applications grows. The Center for Biomedical Informatics and Information Technology (CBIIT) of the US National Cancer Institute (NCI) convened a virtual workshop with the intent of summarizing the state of the art in de-identification technology and processes and exploring interesting aspects of the subject. This paper summarizes the highlights of the first day of the workshop, the recordings, and presentations of which are publicly available for review. The topics covered included the report of the Medical Image De-Identification Initiative (MIDI) Task Group on best practices and recommendations, tools for conventional approaches to de-identification, international approaches to de-identification, and an industry panel.

## Introduction

The Center for Biomedical Informatics and Information Technology (CBIIT) of the US National Cancer Institute (NCI) convened a two half-day virtual workshop in May 2023 for an unrestricted global audience with the intent of summarizing the state of the art in de-identification technology and processes and exploring interesting aspects of the subject. The workshop was organized in eight sessions over 2 days, each session being focused on a specific topic. This summary provides an overview of the content of the presentations and discussions during each of the sessions; the recordings and presentations of which are publicly available for review at http://wiki.nci.nih.gov/display/MIDI/2023+Medical+Image+De-Identification+Workshop. Being a report of events that transpired, it is not intended to be an introduction to the basic principles of de-identification, but rather to address the state of the art and controversial and promising topics of relevance. Readers interested in background material might want to consult some existing recent comprehensive references [1–3].

A brief summary of the topics presented on the first day includes the following:

- Welcome and Introduction
- Report of the MIDI Task Group—Best Practices and Recommendations
- Tools for Conventional Approaches to De-identification
  - The Tools of TCIA—Standardizing Zero-Tolerance De-identification
  - XNAT Platform: Image De-identification
- International Approaches to De-identification
  - Medical Data De-identification—A Canadian Perspective
  - Data Infrastructures For AI In Medical Imaging: A Report On The Experiences Of Five EU Projects
  - Legal Framework and Best Practices for Medical Image De-identification in the EU
- Industry Panel On Image De-identification
  - Introductory Remarks to the Industry Panel
  - Optimizing and Automating Radiology Data De-identification Workflows
  - The Flywheel Platform for Intelligent Image Anonymization
  - Advances in Medical Imaging De-Identification and the Impact of Regulatory Constraints
  - Automated Medical Data De-Identification and Obfuscation
  - Medical Imaging De-Identification On Both Images And Text Using AI Models

## Welcome and Introduction

The session chairs, participants, and attendees were welcomed [4]. The context for the virtual workshop was explained. Many research institutions facilitate, or otherwise require, public sharing of medical images to generate knowledge and gain insights into human health and develop innovative solutions to reduce, or eliminate, disease burden. Patient privacy ethics and laws require that medical images are de-identified before they are disseminated through public repositories (e.g., Imaging Data Commons [5]). In 2020, CBIIT began to engage with the research community on the topic of medical image de-identification by considering and promoting technological solutions. In 2021, a task group was created to provide recommendations for best practices to minimize the risks while preserving the research value of medical images. The goal of this workshop was to convene thought leaders, SMEs, technology developers, and members of the greater biomedical research community to highlight and learn about best practices and innovative technologies in medical image de-identification, discuss questions, and identify challenges. The scope was defined as radiology and pathology images, with the primary emphasis being on DICOM radiology images. For the purpose of the workshop, de-identification was considered to mean de-identification of medical images of human subjects and biospecimens, such that re-identification risk of ethical, moral, and legal concern is sufficiently reduced to allow unrestricted public sharing for any purpose, regardless of the jurisdiction of the source and distribution sites, as defined in the report of the MIDI task group [1].

## Session 1: Report of the MIDI Task Group—Best Practices and Recommendations

The major features of the content of the Report of the MIDI Task Group [1] were summarized [6], with an emphasis on best practices and recommendations of particular interest. The background, mission, goals, charge, scope, and deliverables of the task group were summarized. Best practices (BP) were explained as what you should be doing now, as distinct from recommendations for further research, investigation, development, and documentation. The methodology adopted by the task group was described as involving an extensive though informal literature review as well as monthly discussions of major, difficult, unexplored, or controversial topics. An overview of the content of the report is summarized in Table 1.

**Table 1:** Overview of MIDI Task Group report content

Best practices and recommendations	Image features—derivation of face, age, sex, race from photos, radiography
Scope	Metadata lurking in obscure places—inside JPEG bitstream
Terminology	Modality-specific issues—including external photos, WSI
File formats—DICOM, non-DICOM, standard and proprietary, private extensions	AI used for de-identification (not just as customer for de-identified data)
What needs to be de-identified—within files, in accompanying or linked data sets (e.g., clinical)	Reports, documents, annotations
Rule-based de-identification (emphasis on DICOM PS3.15 profile)	Evaluation, scoring, motivated intruder attack
Statistical Disclosure Control (SDC): re-identification threat model, risk, indirect identifiers +/− modification	Operational and deployment considerations, including scalability, quality control, tools
Structured, unstructured, burned-in, dates	

Time did not permit a complete discussion of all the best practices and recommendations, but selected best practices that were deemed most important were individually discussed and critical aspects were emphasized. What was presented is summarized here, and readers are referred to the complete afore-referenced report for the elided content:

- BP#1 - Everything and quantify risk: “Thorough de-identification by removal or replacement of all known direct and relevant indirect identifiers and sensitive information, … collection descriptions and supporting data, structured and unstructured text data elements, pixel data, and geometric and bitmapped overlays … quantify residual re-identification risk with respect to a pre-determined risk threshold, to justify retention of selected indirect identifiers or sensitive information.”
- BP#3 - Remain compliant: “… should not compromise the conformance of the resulting data … retain DICOM … retain referential integrity … retain functionality."
- BP#4 - Preserve utility: “… preserve as much information about the image acquisition as possible … to maximize the re-use potential, [without] … increasing the residual re-identification risk”
- BP#6 - Use the standard profile: “the current release … of the DICOM PS3.15 E.1 Application Level Confidentiality Profile … knowledge of other unsafe attributes, including private data elements … removing or replacing everything that is known to be unsafe, and retaining only what is known to be safe … options beyond the baseline for retention, cleaning, or removal of information for various scenarios … balance preservation of utility against residual re-identification risk …”
- BP#8 - Non-DICOM: “For non-DICOM images … general principles explicit or implicit in DICOM PS3.15 E.1 should be applied … For clinical data elements … PhUSE De-Identification Standard for CDISC SDTM should be applied”
- BP#9 - All elements anywhere: “… all data elements linked to images in the collection … accompanying spreadsheets or publications … need to be de-identified and subject to a risk analysis … search for the existence of such linked data should be undertaken”
- BP#10 - Burned-in text: “… burned-in text, foreign objects with textual information … discarded or the offending information redacted, manually or automatically (subject to subsequent human review) … not sufficient to limit checks for offending information to only a stratified sub-set of image types …”
- BP#13 - Private elements: “Private data elements retained to preserve utility … reliable source of known safe private data elements … Otherwise, private data elements should be selectively or entirely removed.”
- BP#14 - Obscure metadata: “Compressed bitstreams … should be considered with respect to the potential for identity leakage … scanned for data elements at risk and those selectively removed or replaced …”
- BP#15 - Faces: “… re-identification risk of head and neck cross-sectional images … which may contain potentially reconstructable facial information (PRFI) … humans or facial recognition software … quantified with a realistic collection-specific expert statistical analysis … above a predetermined acceptable risk threshold … facial features removed or modified to reduce the risk … or the images should not be publicly shared.”
- BP#17 - Faces: “… human quality control (QC) … de-identification and preservation of utility … percentage and type of records inspected … guided by a documented risk assessment … process should address structured and unstructured text data elements, pixel data, geometric and bitmapped overlays, and compressed bitstream embedded metadata …”
- BP#18 - Documentation: “The process … used … documented in detail … published with the data collection …”

Examples of selected tools and use cases were shown when it was anticipated that the audience might be unfamiliar with the topic, such as the ARX data anonymization tool [7] and the dicom3tools dciodvfy DICOM validation tool [8].

Since a key best practice is to use the standard DICOM PS3.15 profile [9], a description of the problem statement for the development of the profile and how the profile addresses those problems was provided. The DICOM Standards Committee recognized that in the absence of a standard, nobody would perform de-identification in the same way. There is a need to anticipate and accommodate regulatory requirements, which vary by jurisdiction and may change over time. The committee also considered legal, moral, and ethical requirements. Frequently, a disciplined risk analysis is not performed. Users may be inexperienced, yet without access to consistent or reliable pre-configured tools, they face a relatively complex problem. The DICOM PS3.15 profile solution is to tabulate all potential identifier-containing data elements and describe what action to take for each of them, with specific options to remove or retain more information depending on the utility preservation required. Of critical importance, the profile is maintained to handle new attributes added to DICOM for new use cases and is available in machine-readable form to drive software tools. During the workshop session, the documentation of the standard profile and options was described in some detail. Examples of use cases such as for conventional data elements, structured content, text-based descriptions, and text burned into the pixel data were illustrated. The importance of insisting on using the latest release of a standard like the DICOM PS3.15 profile and only deviating from it cautiously and with a well-informed risk analysis was emphasized.

The problem of obscure metadata, such as in compressed bitstreams, was illustrated using the example of camera settings and GPS location in the EXIF header of a JPEG photograph. Examples of reconstructed faces from CT images were shown.

Selected recommendations, limited by time, were also discussed:

- REC#5 - Quantify performance: “… quantifying the reliability of the de-identification process … ‘scores’ … consumer selecting a process … comparison of different processes … competition or challenge”
- REC#8 - Actual risk of faces: “… actual incremental re-identification risk of potentially reconstructable facial information in head and neck cross-sectional images … realistically assess the need for restricted access … diminished utility of limiting access to, or de-facing such images, especially for head and neck cancer.”

In closing, it was recognized that there may be disagreement over some topics, especially beyond the cancer community members who were the primary contributors. The need to consider unanticipated secondary uses with respect to preserving utility was emphasized.

### Discussion

It was observed that though virtually all medical imaging manufacturers follow the DICOM standard for image production, those who provide medical imaging services have been slow to adopt the DICOM PS3.15 confidentiality profile for de-identification, though many research and open-source tools already support the profile. The reasons for this may include lack of awareness among providers and customers, and this might be mitigated with more education, in addition to working with industrial partners in various projects.

It was asked if we move from the HIPAA Privacy Rule 18 Element (“safe harbor”) method to the expert determination method, based on a statistical risk analysis, is this risk analysis something that can be done one time on a repository basis, or must it be performed for each data set that is submitted to a repository? The safe harbor and expert determination approaches are not mutually exclusive, and the former was developed based on the principles of the latter. The safe harbor has been reexamined in the literature to evaluate its continuing relevance.

For programmatic use of DICOM PS3.15, since 2013, the DICOM standard has been available in XML form, which facilitates the automated inclusion of the profiles and options in updated software tools. Sample extraction stylesheets are provided with the distribution.

The importance of de-identifying images without decompressing and recompressing them in a lossy (mathematically irreversible) way was emphasized; otherwise, image quality may be impaired and qualitative and quantitative diagnostic and analytic tasks may be affected. This also applies to the removal of burned-in text, where one can remove only the blocks where the text is, and not impact the remainder of the image.

The matter of patient age and sex as indirect identifiers was raised, including their recovery from the image data of the body itself, with a finite accuracy that may be comparable to the aggregation that is performed during statistical disclosure control. Sharing of age and sex in the metadata of public image datasets has been a common practice without adverse consequences so far. The importance of some patient characteristics for quantitative analysis (e.g., PET SUV) was noted. Age is important for analysis of some types of cancer, including pediatric tumors. The population is aging so the statistics related to re-identification risk may also be changing, e.g., with respect to the safe harbor over 89 years rule. It was noted that the demographics may be present in clinical data shared by other groups over which one has no control.

Data sharing requirements of funding agencies were mentioned, but were not within the scope of a technical discussion, as opposed to a matter of policy, since the requirements do not specify a particular means of de-identification, risk analysis, or risk mitigation.

Synthetic datasets and generative AI were mentioned but are out of the scope of a technical discussion of de-identification of real data. A comment was made that the threat model to protect against is different for internally shared data than for publicly shared data. Such things as prosecutor risk versus journalist risk, and maximum or average risk, need to be considered in the statistical model.

## Session 2: Tools for Conventional Approaches to De-identification

In setting the stage for this session [10], emphasis was placed on the need for open access or shared research data to comply with regulations that govern patient privacy, including the Health Insurance Portability and Accountability Act (HIPAA) in the USA and the General Data Protection Regulation (GDPR) in the EU. These regulations require the removal of protected health information (PHI) and other personally identifiable information (PII) from datasets before they can be made publicly available. Covered Entities (USA) or Data Controllers (EU) are legally responsible for compliance, even if the data publisher is exempt.

The terminology for De-identification, Anonymization, and Pseudonymization was summarized:

- De-identification of medical record data refers to the removal or replacement of personal identifiers so that it would be difficult to re-establish a link between the individual and his or her data [11]:
  - The removal of specified individual identifiers as well as the absence of actual knowledge by the covered entity that the remaining information could be used alone or in combination with other information to identify the individual (HIPAA, 45 CFR Part 160 and Part 164).
- Anonymization refers to the irreversible removal of the link between the individual and their medical record data to the degree that it would be virtually impossible to reestablish the link.
  - To achieve anonymization under GDPR, re-identification of a data subject must be impossible.
  - Anonymized data is excluded from GDPR regulation altogether because anonymized data is no longer “personal data.”
- Pseudonymization replaces personal identifiers with nonidentifying references or keys so that anyone working with the data is unable to identify the data subject without the key.
  - This type of data may enjoy fewer processing restrictions under GDPR.

The use of DICOM for image exchange and the use of the DICOM profile was described. DICOM is an international standard and is recognized as such (ISO 12052 [12]), so it does apply universally. The importance of the DICOM de-identification profiles [9] was again emphasized since they establish a firm basis for performing de-identification or anonymization. Such profiles are not generally available for other image data formats.

The need for legal agreements, tools, secure transport protocols, and procedures was emphasized. Legal agreements must be in place between the Covered Entity or Data Controller and the Data Processor (Data Publisher). TCIA, for example, has standard legal frameworks for data submitters for use in the USA and Europe. Tools need to be available for identifying, removing, or remapping PHI and PII. Most de-identification tools in the USA focus on compliance with the HIPAA Safe Harbor method rather than the Expert Determination method. Secure data transport protocols must be used for transferring data before it has been de-identified, even if data is mostly or partially de-identified before leaving the submitting site. Additional procedures are required to ensure nothing is missed.

### The Tools of TCIA—Standardizing Zero-Tolerance De-identification

The Cancer Image Archive (TCIA) [13] and its tools were introduced, with a focus on standardized, zero-tolerance de-identification [14]. After a brief overview of the radiology and pathology collections in the archive, and their multiple modalities, multiple file formats, size, and other characteristics, the workflow for submission, curation, and publication was described. Before datasets can be submitted, an approval process is applied, which involves stakeholders from the TCIA Advisory Group, which includes the NCI Cancer Imaging Program, Cancer Diagnosis Program, Center for Cancer Research, and Frederick National Laboratory.

The use of the Clinical Trials Processor (CTP) [15] for processing DICOM images, and the TCIA Submission Wizard to import files, map patients, and anonymize and transfer files, was summarized. Aspera Faspex or Box are used for non-DICOM files.

The curation process for both metadata in the DICOM “header” and the pixel data was illustrated, with reference to a synthetic dataset for testing the process [16, 17]. A brief overview of the Posda [18] tool was provided, highlighting the timepoint architecture, the worker nodes and activity workflows, and the visualization tools, as well as the multi-format and multi-language support. The use of the DICOM PS3.15 de-identification profile [9] and its various options was described, as was the use of the dciodvfy DICOM validation tool [8] to evaluate the conformance of the de-identified objects. The options generally applied include retaining safe private data elements as well as patient characteristics, device identity, data modification with preservation of longitudinal integrity, and cleaning of descriptors, pixel data, and recognizable visual features.

The importance of a visual review of the de-identified result was emphasized. Tools for specific modalities and applications were described, including Kaleidoscope [19] for reviewing an entire series of cross-sectional images “holistically” (all images at the same time) using maximum, average, and minimum projections, the Pathology Visual Reviewer for histopathology whole slide images, and various other viewers such as Quince and OHIF for deeper review and cleaning. To approach zero tolerance, the use of a tabular review tool for the metadata allows human curators to easily review very large curation reports that are sortable, easy to scan visually, and which have suspicious items highlighted, changes confirmed and batch updates to the data performed.

Future potential enhancements to the tools were discussed, including improved file format support, enhanced de-facing tools, improved pathology curation tools, enhanced visualization tools, improved clinical data curation with semantic integration, improved object store support, and improved high-performance computing (HPC) support.

The publication process was briefly summarized. Currently, this uses the National Biomedical Imaging Archive (NBIA) software platform [20] for radiology images and the PathDB/Eaglescope /caMicroscope platform for pathology images.

### XNAT Platform: Image De-identification

An overview of the Extensible Neuroimaging Archive Toolkit (XNAT) platform [21] and its de-identification features was provided [22]. XNAT is an open-source imaging informatics software platform dedicated to helping perform imaging-based research. The core functions of XNAT to manage importing, archiving, processing, and securely distributing imaging and related study data, and the related workflow, were illustrated. Unlike TCIA, XNAT is a platform and not a service, and the nature and level of de-identification is defined by site administrators and project owners. The platform provides for quarantine, local use, collaboration, and public access. It scales from small (single investigator) to very large (federated sites). As a platform, it is distinct from specific repositories like TCIA or IDC.

XNAT can ingest DICOM files through a DICOM C-Store or ZIP upload, from clinical scanners or workstations, departmental researchers, or external collaborators, after which site-wide and/or project-specific de-identification can be performed. Being research-oriented, the data can be segregated according to investigator-specific criteria. An XNAT desktop client, recently released, allows for de-identification on-site before upload. XNAT supports a script-based DICOM editing tool that scrubs and remaps DICOM metadata and can also remove rectangular patches of pixel data. The same software can be run in the desktop client, at the XNAT ingestion boundary, on a site-wide or project-specific basis, or within XNAT triggered by a user action. XNAT has a container service: Docker containers that support various algorithms, including de-facing, can be inserted into the workflow.

A site may deploy multiple XNAT instances configured appropriately to their use. Washington University, for example, has both public and private instances with different practices. Data for internal laboratory-wide research is not scrubbed to the same extent as that which is publicly shared, according to the appropriate threat model.

### Discussion

The matter of how to perform initial de-identification before the data leaves the submitter’s site for non-DICOM data was raised. This is usually addressed on a case-by-case basis as a best effort and is the responsibility of the source site. The risk incurred is a major reason for having a second centralized more robust de-identification and for the protection of the confidentiality of the intermediate material in transit and at rest.

Since Docker was mentioned, the question of the need for a Dockerized version of the Posda tools was identified, and this is available [23].

How much human review of de-identified images is required? It was reiterated that TCIA reviews all images during curation, in order to support their zero-tolerance policy, and actually has two curators look at every image. This is why tools have been developed to improve the visualization process. There is even an additional review on the publication platform, but that is of only a sample, typically 10%, and the purpose is more to verify the integrity of the publishing process. There was interest in the features of the Kaleidoscope for visualization and particularly how the images were stacked and windowed to maximize the likelihood of text detection even if the images themselves were not easily seen. There is also the ability to open suspicious images in other viewers as appropriate. The primary purpose of Kaleidoscope is to quickly detect that there is a problem, which can then be further investigated.

A question was asked about using Posda for bulk de-identification from within Python. Since the package is containerized, it can be started from any language, including Python. Though most of Posda’s scripts are written in Perl, the maintainers are developing more Python script going forward.

It was asked what procedures were used to handle text attributes like SeriesDescription that have valuable content but may also contain PHI. The answer from TCIA was that this is handled manually in the sorted tabular reports of suspect values. It is surprising how quickly curators can handle this process. The private data element knowledgebase helps with this process. For TCIA sharing, SeriesDescription is often replaced entirely with a more meaningful description than the site supplied, given its importance in the user interface. This is a human process and is not automated, and as yet there is not a standard list of values for SeriesDescription, though there are some recent efforts to address this old problem.

It was asked at what point in the process datetime shifting was implemented, and whether or not it occurred before ingested data was quarantined. For TCIA, this is performed at the data submitter’s site, as is UID replacement and pseudonymized identifier replacement using CTP.

It was asked whether a DICOM de-identification challenge could be organized by the Society for Imaging Informatics in Medicine (SIIM) for training purposes. There is currently a plan for such a challenge organized by NCI Medical Image De-Identification Initiative (MIDI) for Medical Image Computing and Computer-Assisted Interventions (MICCAI) 2024, which was discussed on the second day of this workshop.

How to justify the significant costs of de-identification and manual review was discussed. The short answer was that the regulators require it and penalties are significant.

There was discussion of other potential tools that might be used, such as Microsoft Presidio, and the panelists replied that they have no experience with those yet.

There is tension in institutional review boards (IRBs), ethics committees, regulatory compliance, legal risk management departments, and data governance committees between the need to share data and the associated legal risks. An important factor is the requirement of the funding agencies that data be shared. It is also important to be open and share all the documented policies and procedures involved to demonstrate their robustness. International collaborations can be more challenging because of different rules.

The discussion closed with the question of whether complete automation would ever be practical and the human removed from the loop. The consensus was that this was unlikely in the near future. The fact that zero tolerance is difficult to achieve and has significant costs was also discussed.

## Session 3: International Approaches to De-identification

It is well known that there are regional differences in moral, ethical and legal expectations with respect to privacy and the means to assure it. Accordingly, this session attempted to explore some of these differences with a particular focus on the experience in Canada and Europe.

### Medical Data De-identification—A Canadian Perspective

The session began [24] with a review of some of the recent large data breaches of patient records, to emphasize that medical data breaches and privacy issues are an ongoing problem [2, 3]. The recent Information and Privacy Commissioner of Ontario (PCO) Decision 175, which considers the appropriateness of de-identifying personal health information under Ontario’s Personal Health Information Protection Act (PHIPA), was described, emphasizing that de-identification is a permitted use, practices must be disclosed to individuals, and robust security measures are required [25]. The importance of considering the fine details of the decision was emphasized.

The white papers of the Canadian Association of Radiologists on general principles [26] and practical considerations [27] were referenced. The process of acquiring images was reviewed. The terminology used in Canada for de-identification, anonymization, pseudonymization, and encryption was described, with particular emphasis on de-identification being the means of removing from the medical data direct and indirect identifiers. The similarity of the attributes listed in the Canadian Personal Information Protection and Electronic Documents Act (PIPEDA) [28] to those in the HIPAA Privacy Rule “safe harbor” [29] was noted. The need to account for indirect identifiers in combination to re-identify an individual was described. The continuum between identifiable and not identifiable data was illustrated as was the balance between privacy protection and data utility (for secondary purposes) and the concept of an identifiability threshold and an acceptable threshold. The so-called prosecutor risk and journalist risk were distinguished with respect to assessing the risk of re-identification, that being the risk of matching to a real person and by doing so learning something new about that person.

Next, the overall process of ingesting and de-identifying data at a typical institution was reviewed. The various types of data records and how they might be indexed and located without compromising patient privacy were discussed. Once a cohort of data has been defined, the process of de-identification was described. Particular aspects including hand-writing and burned-in text redaction, date shift and medical record number consistency, and differential privacy were emphasized, as was the importance of protecting and restricting access to any lookup table used for pseudonymization and data shifting over time. Uses of de-facing and burned-in text redaction were illustrated, as was the use of locally trained natural language processing (NLP) models of free text medical reports, to allow for the extraction of information without compromising privacy. The concepts of differential privacy and k-anonymity as applied to tabulated indirect identifiers were demonstrated. Handwriting redaction was also considered. Finally, it was emphasized that true healthcare improvements will occur with increased mobility and movement of medical data and that privacy does not need to be compromised to accomplish this, given that technology exists to export, aggregate, and de-identify medical data almost instantly. More organizations need to adopt these tools to empower innovation.

### Data Infrastructures for AI in Medical Imaging: A Report on the Experiences of Five EU Projects

The activities of the Data Management Working Group of the AI for Health Imaging Network (AI4HI) in Europe in reviewing five European Union (EU) projects involving de-identification were described [30]. The projects were as follows:

- EuCanImage—A European Cancer Image Platform Linked to Biological and Health Data for Next-Generation Artificial Intelligence and Precision Medicine in Oncology
- CHAIMELEON—Accelerating the lab-to-market transition of AI tools for cancer management
- INCISIVE—A multimodal AI-based toolbox and an interoperable health imaging repository for the empowerment of imaging analysis related to the diagnosis, prediction, and follow-up of cancer
- ProCAncer-I—An AI Platform integrating imaging data and models, supporting precision care through prostate cancer’s continuum
- PRIMAGE—PRedictive In-silico Multiscale Analytics to support cancer personalized diagnosis and prognosis, Empowered by imaging biomarkers

The key dimensions of the data infrastructures involved have recently been compared [31]. Different cancer types, numbers of subjects, architectures, data models, formats, de-identification processes, and curation tools were involved. In addition to images, associated clinical data is gathered in most of these projects. The projects are distinguished by whether or not they aim for pseudonymization or anonymization.

The CHAIMELEON de-identification process involves separate steps of pseudonymization and anonymization. For pseudonymization, the DICOM PS3.15 profile [9] is used, with clean descriptors, retaining full dates and patient characteristics options, and direct identifiers which are either removed or replaced by a randomly generated pseudonym. A table of correspondence is kept securely within the hospital, and clinical data are associated with images using the same pseudonym. All dates, including study dates and dates of birth, are kept at this stage, for handling discrepancies during the curation process. In the anonymization step, data curators perform a quality check, a new patient identifier is generated (for which no table of correspondence is kept), all dates are shifted to maintain the integrity of longitudinal information, and data is sent to the central repository.

The EuCanImage de-identification process entails pseudonymization of DICOM images in a single step to encrypt the patient’s medical record identifier in a cloud-based GDPR-compliant platform using a truncated SHA512/256 hash. Input information is a secret key (unique for each hospital) concatenated together with the personal patient’s ID, and the final patient’s hashed identifier is a 64 alphanumeric character code. Clinical data is collected separately, using an electronic case report form (eCRF) [32]. The data attributes collected do not contain any direct identifier, only patient pseudonyms. Additional measures are enforced to decrease the risk of identifiability, such as replacing the date of diagnosis by age at diagnosis, substituting the collection of dates of starting and ending of procedures by periods of time when possible, or the use of arbitrary dates.

The INCISIVE de-identification process uses the CTP Anonymizer [15] with a custom protocol for anonymizing DICOM images, established collaboratively between data providers, AI developers, and legal partners, finding the balance between the usability of the data by the AI developers and privacy. For the name and identifier of the patient, a naming convention is used. For other identifiers, a hash function is applied using the de-identified patient identifier as a seed. For the dates, the original offset between consecutive studies of the patient was preserved after the de-identification process. Other DICOM fields that might contain information leading to patient identification and were not useful for the AI developers were either removed completely or replaced with a zero-length value. In a second step, the project developed their own de-identification tool, based solely on the DICOM PS3.15 profile [9], and which gives the data providers the option to select the level of privacy they want to apply to their data by various options inside the tool.

The ProCAncer-I de-identification process again uses the DICOM PS3.15 profile [9] and the CTP Anonymizer [15], with a set of rules in a designated script file. Dates were shifted by an offset defined from the original study date, and other extreme accuracy fields such as time of acquisition were obscured.

In the PRIMAGE de-identification process, access is granted to several registries and clinical trial databases for secondary use of available clinical data. When a new patient is incorporated in the database, a new and unique pseudonym is given. All the DICOM tags with sensitive information as stated in the DICOM PS3.15 profile [9] are removed or emptied from the uploaded files.

The new EUropean Federation for CAncer IMages (EUCAIM) [33] was then introduced, which aims to address the fragmentation of existing cancer image repositories.

### Legal Framework and Best Practices for Medical Image De-identification in the EU

This presentation from Aigora [34] provided a brief overview of the EU regulatory framework, pointed out differences between GDPR [35] and HIPAA Privacy Rule [29] with respect to de-identification, and shared at a high level practices employed to achieve and preserve medical image de-identification. Multiple layers of regulation in Europe were mentioned, including those applicable at the EU, national, state, institutional, and individual physician levels. GDPR is generally applicable, as the de-identification procedure itself constitutes a processing of personal data to which the data protection regulation applies. In addition, peculiarities of national data protection legislation need to be considered, and further restrictions may apply, varying by state and clinical setting.

The definitions of and requirements for de-identification under GDPR and HIPAA were compared. It was emphasized that there is no prescriptive standard for de-identification in EU legislation; unlike HIPAA, GDPR does not clarify the approach, but rather the focus is on the outcome. HIPAA specifies two approaches, the expert determination and so-called “safe-harbor” methods, whereas GDPR focusses on minimizing the residual risk of re-identification, taking into account “means [that] are reasonably likely to be used … costs … and … time required for identification … available technology at the time of the processing.” Under GDPR, several anonymization techniques may be envisaged. Both technical and non-technical measures may be considered for medical image de-identification while weighing re-identification risk against data utility. In guidance by the European advisory body on data protection [36], the optimal solution should be decided on a case-by-case basis, evaluated based on the possibility of still singling out an individual, linking records relating to an individual, or inferring information concerning an individual; the severity and likelihood of residual risk of re-identification linked to any anonymization technique needs to be assessed.

Best practices in use by Aigora to achieve de-identification of radiology images include rule-based and statistically based de-identification, e.g., removal or replacement of DICOM “header” elements, application of hashing techniques, data aggregation, and k-anonymity. Further to this, removal of burned-in and unstructured text as well as de-facing is used where applicable and possible. Then there is always a manual quality control step. Measures to preserve de-identification include technical and organizational measures as well as contractual agreements and structural layers (including blinding to the original source of the data).

In addition to the foregoing, key takeaways and recommendations included the recognition that some residual risk of re-identification will always remain. In light of a need to balance data minimization with data utility, one should also consider alternatives to anonymization, such as pseudonymization, generally requiring patient consent and ethics committee approval.

### Discussion

Given that the residual risk of re-identification is always non-zero, what threshold should be used? The 5% threshold, such as is defined for use in Canada, and how that might be established and justified, was discussed. GDPR does not say 0%, leaving room for interpretation.

## Session 4: Industry Panel on Image De-identification

### Introductory Remarks to the Industry Panel

The session began with an introduction to the need for responsible and equitable application of data science and technology especially in the field of artificial intelligence machine learning. The importance of industry participation and collaboration with academia in order to make tangible progress was emphasized. Five industry leaders in the field were introduced [37].

### Optimizing and Automating Radiology Data De-identification Workflows

The work of Impact Business Information Solutions (IBIS) was presented [38] with respect to reducing the requirement for a human in the loop, particularly as the need for more data to feed AI machine learning applications is growing. Current de-identification solutions require a substantial human review component, which is both expensive and a constraint on data throughput. Some default de-identification solutions can be overly aggressive, unnecessarily reducing the utility of the data for secondary use. A solution must satisfy basic needs for user-friendly user interface (UI) with configurability, ease of deployment, and scalable performance, both horizontally and vertically.

Working with academic partners, IBIS is developing a highly configurable, extensible platform for automation of the deidentification process with minimal need for human review. The approach combines rule-based de-identification with AI-based de-identification into configurable workflows/pipelines. The rule-based component is configurable and template-based, intended to match existing solutions in the public domain. The AI-based component includes modality-specific algorithms, including models specific to MR, CT, PT, US, and X-ray, as well as a generalized model. The approach uses a hub-and-spoke architecture providing for cloud-based or enterprise-based command, configuration, and control, with execution at the edge, adjacent to the data, all within the context of regulatory compliance. The solution automates the execution of de-identification pipelines and audits all user activity and pipeline execution. The next phase of the project will include an AI-based uncertainty quantification algorithm, to more accurately inform decisions about the need for human review.

Next, an overview of the architecture of the EICON REACH de-identification platform was presented, explaining the relationship between the hub and the clinical access nodes (CANs), the latter located adjacent to the data to be de-identified. Execution of a typical pipeline was shown. The images are ingested, the metadata is parsed and normalized, and the rule-based de-identification is applied to the metadata, and then using the appropriate modality-specific trained model, the regions of interest in the image are predicted and extracted. Optical character recognition (OCR) is then performed to extract the text from the images, which are subjected to NLP and redaction of identifying information. Basic internal metrics are used to drive a decision to submit the de-identified images for human review. This will be augmented in a later phase with uncertainty quantification. This de-identification approach is not just a standalone approach but can be integrated into broader clinical workflows and use, for example, for pseudonymous transport of clinical images for cloud-AI processing and then re-identification of the results on-site for return to the clinical PACS.

### The Flywheel Platform for Intelligent Image Anonymization

**Fig. 1:**
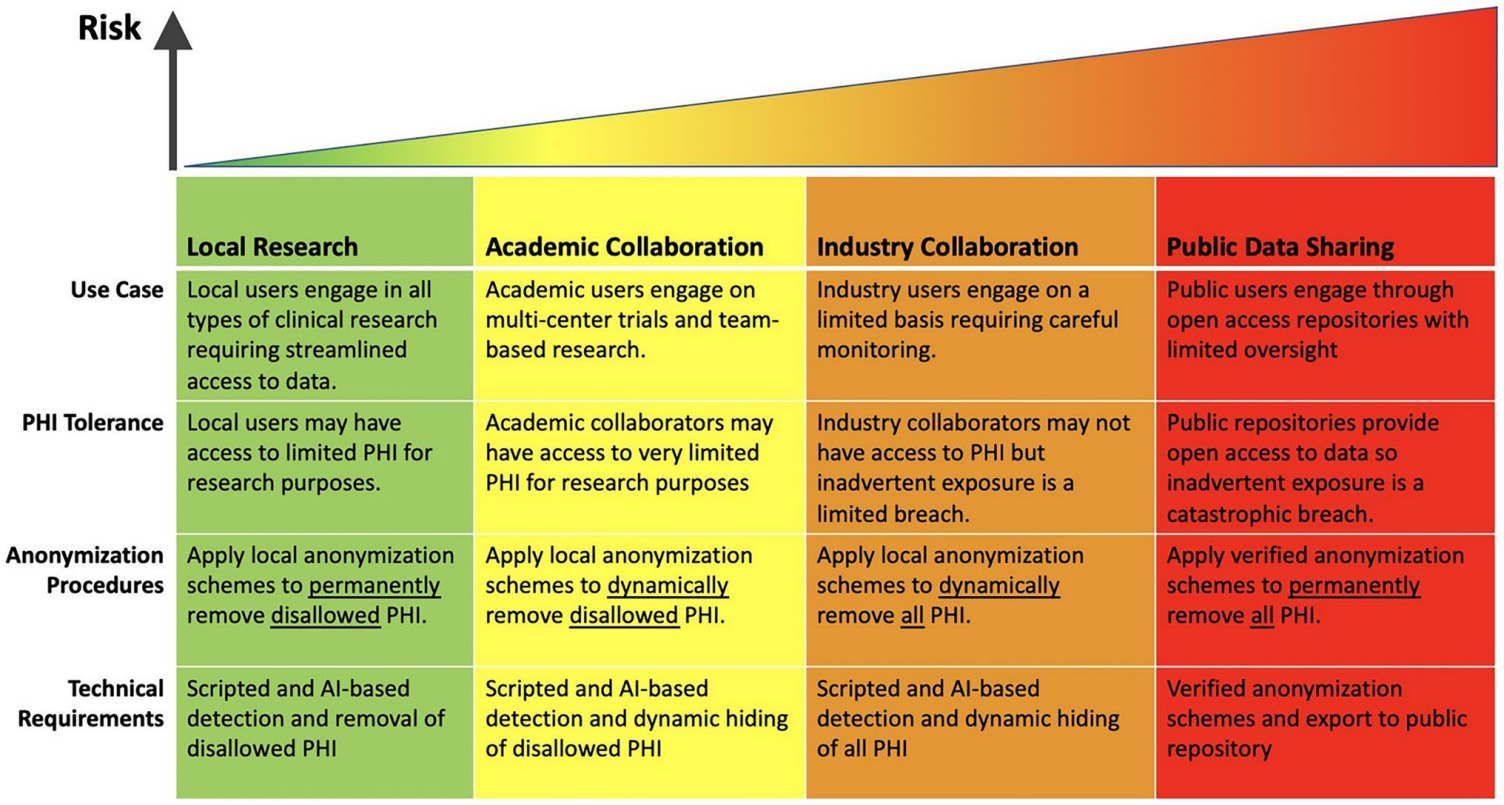
Risk versus use-case, tolerance, procedures and technical requirements

The work of Flywheel, a medical imaging data management and collaboration platform that supports getting data from modalities, was presented [39]. De-identification is performed at multiple steps in the pipeline including during capture, curation, data collaboration, and data sharing initiatives. An overview of different applications and use cases was provided, both on-site and in the cloud, at various scales. The relationship to various open-source initiatives was described, including XNAT and various open data archives and data compute libraries. The existing de-identification capabilities of XNAT [40] and Flywheel [41] were referenced. The NCI SBIR Phase II “Intelligent Image Anonymization with XNAT” awarded to Radiologics, now operating under the Flywheel banner, was described. The problems to be addressed include text-based information in file metadata and the image pixels, facial features inherent in neuroimaging, and other features in the images. What has changed over the last 20 years include larger data sets, more available data, NIH data sharing requirements, and more creative computing approaches (e.g., federated learning). A lot of the tools that require manual review were really built for smaller datasets in the tens or maybe hundreds. As we work with data volumes that are larger by orders of magnitude, the automation needs to be substantially more efficient. A matrix illustrating the continuum of risk versus use case, tolerance, procedures, and technical requirements was presented (see Fig. 1). Existing script-based data element level and series-level configurations were shown. Tools for de-identification review at a large scale were discussed. Techniques for handling an enormous number of images were described, including those for well-known attributes (as in the DICOM PS3.15 profile [9]), heuristics (such as, if an attribute has the same value across a large number of patients in a cohort, it is probably not identifying information), OCR and NLP. The latter was exemplified using an iPhone screenshot to extract text from a medical image and Chat GPT to determine whether or not it was identifying information. The challenges of recognizing unusual names were mentioned.

### Advances in Medical Imaging De-identification and the Impact of Regulatory Constraints

The work of AG Mednet in de-identification for support of multi-center clinical trials was presented [42]. The importance of minimal training requirements for users with little expertise at thousands of sites globally was emphasized, even though individually they may be de-identifying a small number each. The importance of compliance with GDPR and HIPAA Privacy Rule and other regulatory requirements for this use-case was noted. The state of the art in pixel de-identification of DICOM images was described with reference to a tool to delineate identifying information by region and applying pixel removal across multiple similar images. This can be problematic if images within a series are misaligned and may require individual region redaction by a user, which is time-consuming.

All of the operations involving pixel de-identification have to take place on the submitting site side before the images leave the site (cross the firewall). This needs to be done without having to install any software on the trial coordinator’s computer, since generally the user is not able to install software on a computer in the hospital system. In the past, this was done through Java Web Start [43], but now, it is all done in the web browser (which has limitations). Ultrasound and angiography images, which are often compressed, present a challenge. This may require decompression in the browser, and selective redaction techniques that are lossless [44] may be particularly challenging to implement in the browser.

Two possible next-generation approaches are in the prototype stage. One is to define modality formatting, that is, to use the manufacturer and model information to pre-define regions where the identifying information may be and automatically remove these regions. The other is to use machine learning (ML) and large language models (LLMs) to find text in images and remove names. Prototyping with AWS Rekognition [45] has been implemented. This requires uploading the images that contain identifying information. Harnessing AI and LLMs for de-identification of DICOM images can leverage trained models for both pixels and structured reports. Operationally, these models can be deployed in either a computer-assisted or autonomous manner; fully autonomous capability is not yet here and will not be for a little while. Scalability and adaptability of AI solutions to handle large datasets and various medical imaging formats are important considerations as is time. Anything that takes more than a minute or so and involves user interaction is problematic. Some images, such as videos, may take more time.

#### Automated Medical Data De-identification and Obfuscation

The deployment of John Snow Labs’ Healthcare NLP software package for de-identification cases was presented [46]. This solution is designed for enterprise deployment, whether on-premise or in the cloud. It can process a range of document and image formats, accommodate multiple national languages, operate on both unstructured and structured data elements, and offer masking (substituting identifying tokens with a consistent string) and obfuscation (replacing with a random token of equivalent form).

The procedure encompasses the extraction of information from the input document or image, including DICOM formats. This also covers data within embedded contexts, like within PDFs, and image pixel data. The extracted text is then vectorized by the contextual embedding of the BERT type or less resource-intensive non-contextual embedding. Named entities are identified using a modified Bi-LSTM approach. For a comprehensive breakdown of the implementation, refer to [47]. Subsequently, de-identification is executed. The enhancement in performance after integrating regular expressions and a contextual parser was noted, witnessing a 10% boost. The most significant enhancements were seen in the age and location entities. The overall accuracy on a standard benchmark surpassed 95%. When considering only binary classification (PHI vs. no-PHI), accuracy exceeded 98%. Comparative benchmarks for different entities and tools are provided in [48].

Challenges associated with maintaining obfuscation consistency were explored. These include ensuring name consistency (uniformly altering a name in multiple instances for the same subject), maintaining gender congruence in name mapping, age consistency within a given range, clinical consistency (e.g., preserving gender relevance for medical histories like breast cancer), day shift consistency, preserving date format post-shifting, and maintaining text length consistency.

### Medical Imaging De-identification on Both Images and Text Using AI Models

The work of Google in the field of healthcare data de-identification was presented [49]. De-identification essentially consists of two steps: the identification (detection) of identifying information and then the transformation of that information.

For detection, rather than choosing specific DICOM data elements, the approach used is to classify information into specific types. The 18 elements from the HIPAA Privacy Rule [29] are most relevant. AI models for NER are used to extract the type. This is performed in context on the entirety of the text (e.g., the entire value of a DICOM Study Description data element). This approach may have false positives (e.g., classifying “MR HEAD” as “Mr. Head” the person as opposed to the modality and anatomic location). Google is also experimenting with using LLMs for this application, but so far has encountered issues with the results being non-deterministic, but their additional capabilities for edge cases may be advantageous.

Transformation options include removing the offending text entirely, replacing it with a mask of fixed characters, replacing it with the type of data that it represents (using a fixed label), and for specific types, date shifting (preserving chronological order) and hashing.

Pixel-level de-identification using OCR on DICOM images is also supported., and detected text is handled in the same manner as text in other DICOM data elements.

The pipeline is cloud-based and scalable, to petabytes of images.

Associated clinical information is also important for research, so the same approach is applicable to FHIR [50] resources as well.

### Discussion

The panelists were asked about their opinions of AI and LLMs for de-identification of medical images. The probabilistic nature and increased cost of LLMs was emphasized (especially for very large numbers of nodes); on the other hand, they are very flexible, especially for detecting odd types of identifying information, and locally specific variations, such as acronyms. So far, the current performance of LLMs has benchmarked lower than conventional AI models, but their use as an additional layer after rule-based and AI-based approaches have been applied may be useful. Detecting text in images is perhaps more important than classifying that text, since when in doubt it may be discarded, since it is likely not that important. Uploading identifiable information to cloud solutions needs to be considered from a regulatory perspective. Another factor is the use of self-modifying software with continuous training in a regulated environment, particularly the GxP regulations that apply for commercial clinical trials. This leads to a need for controlled versioning and particularly applies to LLMs trained for local specifics.

The scalability of automated solutions while minimizing the human-in-the-loop burden was discussed, in the context of the lack of perfect performance of automated solutions. Uncertainty quantification has been demonstrated to be effective in other vertical applications and may be applicable. Existing models may be able to report quantification of their confidence, but this may not be sufficient. Another approach is to maximize the performance of human review by providing tools for aggregating content.

Unusual image features, including tattoos, barcodes, and pacemaker serial numbers, need to be considered. These are not necessarily in the form of text (an extreme example being faces). The integration of applications from non-medical applications (such as barcode reading and facial recognition) may be integrated. This may require flexibility of configuration at the local site.

Still, even the primary, non-fringe use case has not been entirely solved and should be the primary focus.

The majority of panelists reported that they had already implemented the DICOM PS3.15 profile [9]. A caveat is that individual sites may insert identifiable information into unexpected data elements, which undermines any rule-based approach. There are also other caveats.

Who is liable for a de-identification failure? The service providers indicated that they only provide tools and that the operators (users) remain responsible, especially for configurable solutions.
